# Role of Connexin-Based Gap Junction Channels in Communication of Myelin Sheath in Schwann Cells

**DOI:** 10.3389/fncel.2019.00069

**Published:** 2019-03-01

**Authors:** Bruno A. Cisterna, Pablo Arroyo, Carlos Puebla

**Affiliations:** ^1^Escuela de Medicina, Universidad de Talca, Talca, Chile; ^2^Centro para el Desarrollo de la Nanociencia y Nanotecnología (CEDENNA), Universidad de Santiago de Chile, Santiago, Chile; ^3^Facultad de Medicina, Pontificia Universidad Católica de Chile, Santiago, Chile; ^4^Instituto de Ciencias Biomédicas, Facultad de Ciencias de la Salud, Universidad Autónoma de Chile, Santiago, Chile

**Keywords:** connexins, gap junction channels, myelin sheath, Schmidt-Lanterman incisure, Charcot-Marie-Tooth disease, CMTX1

## Abstract

Peripheral nerves have the capacity to conduct action potentials along great distances and quickly recover following damage which is mainly due to Schwann cells (SCs), the most abundant glial cells of the peripheral nervous system (PNS). SCs wrap around an axonal segment multiple times, forming a myelin sheath, allowing for a significant increase in action potential conduction by insulating the axons. Mature myelin consists of compact and non-compact (or cytoplasmic) myelin zones. Non-compact myelin is found in paranodal loops bordering the nodes of Ranvier, and in the inner and outermost cytoplasmic tongues and is the region in which Schmidt-Lanterman incisures (SLI; continuous spirals of overlapping cytoplasmic expansions within areas of compact myelin) are located. Using different technologies, it was shown that the layers of non-compact myelin could be connected to each other by gap junction channels (GJCs), formed by connexin 32 (Cx32), and their relative abundance allows for the transfer of ions and different small molecules. Likewise, Cx29 is expressed in the innermost layer of the myelin sheath. Here it does not form GJCs but colocalizes with K_v_1, which implies that the SCs play an active role in the electrical condition in mammals. The critical role of GJCs in the functioning of myelinating SCs is evident in Charcot-Marie-Tooth disease (CMT), X-linked form 1 (CMTX1), which is caused by mutations in the *gap junction protein beta 1* (*GJB1*) gene that codes for Cx32. Although the management of CMT symptoms is currently supportive, there is a recent method for targeted gene delivery to myelinating cells, which rescues the phenotype in KO-Cx32 mice, a model of CMTX1. In this mini-review article, we discuss the current knowledge on the role of Cxs in myelin-forming SCs and summarize recent discoveries that may become a real treatment possibility for patients with disorders such as CMT.

## Myelinating Schwann Cells

The ability of peripheral nerves to recover quickly after damage is mostly due to the plasticity of Schwann cells (SCs; Boerboom et al., 2017). SCs are the principal glia of the peripheral nervous system (PNS; Fehmi et al., 2018). In the development of vertebrate PNS, SCs are derived from neural crest cells that differentiate into SC precursors and then into immature SCs (Jessen and Mirsky, 2005; Boerboom et al., 2017). The process of myelination begins around birth, where SCs cover axons that are larger than ~1 μm in diameter (Fehmi et al., 2018).

Myelinating SCs allow for the fast conduction of action potentials by the axons (Jessen and Mirsky, 2005; Boerboom et al., 2017). Each myelin internode is flanked by the nodes of Ranvier, which are bare patches of the axon plasma membrane that are enriched in voltage-gated Na^+^ channels (essential for saltatory conduction). In turn, K^+^ channels are clustered at the juxtaparanodal region of the axon. Thus, the two primary types of ion channels are separated by the paranode (Poliak and Peles, 2003).

An essential process in a functional interaction between axons and SCs, is myelin formation. The molecules that mediate axonal–glial interactions along the internode have been described (Trapp, 1990; Martini and Carenini, 1998; Maurel et al., 2007; Hayashi et al., 2008; Chen et al., 2016). The myelin-associated glycoprotein (MAG), a cell adhesion molecule member of the Ig-superfamily—expressed by myelinating SCs and oligodendrocytes [equivalent to the SCs but located in the central nervous system (CNS)]—has been localized in the internode (Trapp, 1990; Maurel et al., 2007). This protein interacts with several axonal components (Hannila et al., 2007), and it is crucial for the maintenance of myelin, evidenced by the degeneration of myelin and axons in MAG-deficient mice (Martini and Carenini, 1998). In the later stages of myelination, MAG is also found in Schmidt-Lanterman incisures (SLI; Maurel et al., 2007). Additionally, connexin 32 (Cx32) is another component type involved in the maintenance of myelination, which does not belong to the Ig-superfamily (Martini and Carenini, 1998).

Recently, it was reported that nectins and nectin-like molecules (Necl) are required for the axon–glial interactions along the internode through heterophilic interactions. In particular, for cell-cell context, both neurons and SCs express different sets of Necl proteins: axons express Necl-1 and Necl-2, and SCs express Necl-4 and some Necl-2. The main binding protein detected between axon and SCs was Necl-1 and Necl-4 (Maurel et al., 2007). Necl-1 activity involves the PI3 kinase/Akt signaling cascade (Chen et al., 2016). Moreover, Necl-1, Necl-2, and Necl-4 are also expressed at high levels in the SLI of myelinating SCs (Maurel et al., 2007).

## The Issue of Intracellular Communication in Myelinated Schwann Cells

It has been proposed that the number of myelin lamellae in the sheath is directly proportional to the axonal circumference, for example, over the range of 10–80 lamellae in the sciatic and vagus nerves of mice (Friede and Samorajski, 1967), or 18–24 in the optic nerve in humans (Friede and Hu, 1967). A significant amount of myelin membrane has been proposed to increase the axonal membrane resistance and decrease the capacitance, increasing the conduction velocity. According to some estimates, myelinating SCs may produce up to 20 mm^2^ of compacted membrane around the largest axons, about 2,000 times more than typical epithelial cells (Kidd et al., 2013). This massive amount of membrane, concentrated in a small area is a challenge for the communication of the cell itself. The myelinating SCs manages this issue by separating the myelin in two distinct domains, compact myelin, and non-compact (or cytoplasmic) myelin (Kidd et al., 2013).

Compact myelin is a compact membrane spiral, with a periodicity of 13–18 nm per turn (Fernandez-Moran and Finean, 1957; Kirschner and Sidman, 1976), which begins and ends with the apposition of the SC plasma membrane against itself to form the inner mesaxon and the outer mesaxon. Compact myelin has a high (~70%) lipid content and is enriched in galactosphingolipids, saturated long-chain fatty acids and cholesterol (Saher and Simons, 2010), while it has a low protein content with a very low protein diversity; mainly P0 protein, maltose-binding protein (MBP), and peripheral myelin protein 22 (PMP22). Non-compact myelin is in paranodal loops (cytoplasmic extensions located in the lateral margins of the SCs, bordering nodes of Ranvier), and in SLI (continuous spirals of overlapping cytoplasmic expansions within areas of compact myelin), and in the inner and outermost cytoplasmic tongues. While, compaction of myelin removes the aqueous components of the cytoplasm, favoring electric conduction but hindering cellular processes such as intracellular communication, non-compact compartments concentrate the organelles and the necessary components for the cellular operation.

A pioneering observation of the myelin sheath, made using freeze-fracture electron microscopy, reported gap junction channels (GJCs)-like structures in the membrane of the mesaxons, paranodal loops and SLI (Mugnaini et al., 1977; Sandri et al., 1977; Bertaud, 1978; Tetzlaff, 1982). Likewise, the diffusion of fluorescent dyes across the cytoplasmic layers, primarily at SLI, suggest that GJCs provides a direct radial pathway for the transport of ions and small metabolites across the myelin sheath (Balice-Gordon et al., 1998). Then, considering that the unrolled myelin sheath is more than 4 mm long, while the compact myelin sheath is less than 4 μm thick (Friede and Bischhausen, 1980), this potential radial pathway would be more than 1,000-fold shorter than the circumferential pathway (Scherer et al., 1995; Kleopa et al., 2010). For diffusion time, it was proposed that the radial pathway is one million times faster than the circumferential pathway, because, in theory, the diffusion time in a plane is proportional to the square of the distance (Balice-Gordon et al., 1998). However, this investigation has some limitations, for example, the authors assumed that the ions showed a very slow velocity in circumferential diffusion, but they did not measure this pathway nor did they measure the rate of diffusion within the incisures. It is therefore difficult to determine that the radial pathway will be x-fold faster than the circumferential pathway, in fact, the most important real contribution is providing functional evidence, i.e., injection of sciatic nerve fibers, that GJCs mediate a radial pathway.

## Gap Junction Channels in Myelinating Schwann Cells

GJCs connect adjacent cells (heterocellular) and isolated segments of the same cell (autocellular) and allow the diffusion of ions and small molecules across apposed cell membranes expressed in vertebrates (Sáez et al., 2003a). The single channel is formed by the interaction of two apposed hemichannels (HCs) or connexons, each of which is composed of six connexins (Cxs) arranged radially around a central pore (Sosinsky, 1996; Unger et al., 1997). It is worth mentioning that, Cx HCs are semipermeable channels that may allow the passage of small molecules such as ATP (Wang et al., 2013), IP3 and cAMP (Hernandez et al., 2007), NAD^+^ (Bruzzone et al., 2001), and glutamate (Sáez et al., 2003b; Ye et al., 2003), and the passage of ions driven by their electrochemical gradients (Sáez et al., 2005). Cxs are transmembrane proteins that belong to a multigene family with 20 and 21 members in the mouse and human genome, respectively (Söhl and Willecke, 2003). Each Cx HCs can be formed by a single type of Cx (homomeric) or possibly by two or even three Cxs (heteromeric). The GJCs formation can then give rise to homotypic or heterotypic intercellular channels, when they are the same or different Cxs in apposed cells (Jiang and Goodenough, 1996; Koval et al., 2014). Every one of these channel combinations displays different permeability properties and are regulated differently (Weber et al., 2004). Thus, Cxs diversity provides essential variations in cell-to-cell communication.

The presence of the Cx32 is well documented by immunocytochemistry in inner mesaxons, at paranodal loops and SLI of the myelin sheath (Bergoffen et al., 1993a; Scherer et al., 1995; Spray and Dermietzel, 1995). Likewise, these findings were confirmed by freeze-fracture replica immunogold labeling (FRIL). Cx32 is also present at the two outer layers of myelin, suggesting that Cx32 forms GJCs between the non-compact layers of the SCs myelin sheath (Meier et al., 2004).

The Cxs play a critical role in the functioning of the myelinating SCs. Indeed, mutations in the human gene* gap junction protein beta 1* (*GJB1*), which encodes Cx32, lead to the pathological phenotype of the X-chromosomal form of Charcot-Marie-Tooth (CMT1X or CMTX1), where inflammatory processes in peripheral nerves decrease conduction velocity of action potentials, leading to muscle atrophy (Bergoffen et al., 1993a; Fischbeck et al., 1996; Abrams et al., 2002). This disease is the second most common form of hereditary motor and sensory neuropathy, and there is no cure (Kleopa et al., 2012). Despite the abundant evidence that relates the absence or altered function of Cx directly to CMT1X, the role of Cxs in myelin physiology remains poorly understood.

Cx32 has multifaceted functions described in glial cells in the CNS (Abrams, 2017) and PNS (Bortolozzi, 2018). Recent evidence indicated the expression of functional Cx32 HCs that release ATP during electrical stimulation on mice sciatic nerves, which from cultured SCs, by depolarization evoked through a high extracellular potassium concentration (Nualart-Marti et al., 2013). ATP release was then significantly decreased after the sciatic nerve was treated with HCs inhibitors (octanol or carbenoxolone) or after silencing Cx32 from cultured SCs, suggesting that purinergic mediated signaling could contribute to intracellular communication (Nualart-Marti et al., 2013). On the other hand, Ca^2+^ waves do not seem to utilize GJCs in glial cells of the brain. Instead, Ca^2+^ waves are propagated by an alternative extracellular mechanism, involving ATP release and possibly requiring Cx HC activity (Bennett et al., 2003; Nedergaard et al., 2003), suggesting that Ca^2+^ waves may be propagated by an ATP-induced ATP release mechanism. However, the functions of HCs in myelinating SC still needs to be demonstrated. The Cx32 is even involved in the proliferation of SCs, related to neuregulin-1, which does not involve Cx32-mediated intercellular communication (Freidin et al., 2009). Likewise, mice lacking Cx32 display features of CMTX, such as onion bulb formation and slow nerve conduction in myelinating SC (Anzini et al., 1997; Scherer et al., 1998). Moreover, cultured SCs, from Cx32-null mice, are still electrically coupled and even allow diffusion of fluorescent dye (Zhao et al., 1999), implying the presence of other Cxs forming gap junctions (Balice-Gordon et al., 1998).

Connexin 29 (Cx29), a 29-kDa protein (and equivalent to human ortholog Cx31.3; Söhl and Willecke, 2003) was also found in the myelin sheath, by freeze-FRIL, but only in the innermost layer, in close association with the hexagonally arranged intramembrane particle (IMP) “rosettes” in axolemma of large myelinated axons (Li et al., 2002). Similarly, through immunofluorescence (IF), Cx29 was localized in the innermost layer of the myelin sheath, in the paranode and juxtaparanode, tightly colocalized with K_v_1.2 channels in the axolemma (Altevogt et al., 2002). Recently, through IF and FRIL, K_v_1.1/K_v_1.2 channels were identified within the axonal rosette in rodent sciatic nerve and the molecular coalignment 1:1 with Cx29 was proposed to form a xenotypic channel (Rash et al., 2016). On the other hand, K^+^ conductance occurs neither in the perinodal, nor in the submyelinic extracellular spaces, as previously envisioned; it is now proposed to occur in the innermost myoplasm of SCs, which is electrically isolated by the myelin plasma membrane (Rash, 2010). Likewise, the proposed K^+^ recycling from axoplasm to myoplasm to axoplasm could be energetically cheaper than using Na^+^-K^+^ ATPase to axoplasmic replacement. This axon-to-glia linkage implies that myelin may play an electrically active role that underlies both the faster axonal repolarization and the faster conduction velocity of mammalian myelinated axon. However, Cx29 does not prevent the development of demyelinating neuropathy in Cx32-null mice (Scherer et al., 1998), or in Cx32 mutant mice (Jeng et al., 2006; Sargiannidou et al., 2009). Although Cx29 may form HCs (Ahn et al., 2008), these Cx proteins do not form GJCs (in Xenopus oocytes or N2A cells), but at least *in vitro*, it may modify the properties of Cx32 GJCs (Altevogt et al., 2002).

Other Cx proteins, such as Cx26, Cx43 and Cx46, have also been reported to be expressed in SCs (Chandross et al., 1996a,b; Yoshimura et al., 1996; Mambetisaeva et al., 1999; Li et al., 2007), but there are no published data that currently establishes any of these Cxs in SCs. In particular Cx43, together with Cx29 and Cx32 are present during SCs development in mice and displayed a consecutive onset of expression (Li et al., 2007). Similarly, it has been suggested that Cx43 and Cx46 could coordinate cellular responses of SCs after a peripheral nerve’s injury (Chandross et al., 1996a,b).

**Figure 1:**
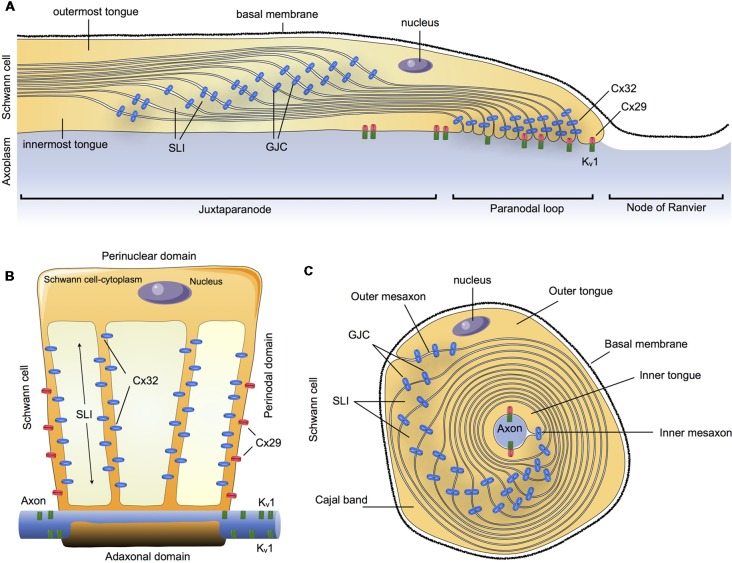
Schematic summary of connexins (Cxs) in the communication of myelin sheath in Schwann cells (SCs).** (A)** Longitudinal view of myelinated SCs showing the different compartments: outermost tongue, innermost tongue, Schmidt-Lanterman incisures (SLI) and the distribution of the connexins forming gap junction channels (GJCs) or hemichannels (HCs) formed by Cxs (Cx HCs) along the juxtaparanode, paranodal loop or the node of Ranvier. **(B)** Myelinated SCs unwrapped from the axon it invests. **(C)** Transversal view. Cx29, connexin29; Cx32, connexin 32; K_v_1, Potassium voltage-gated channel subfamily 1.

In summary, Cx32 and Cx29 are the main Cxs detectable in myelinating SCs in an asymmetric subcellular distribution, with a partial overlapping distribution. Cx32 was found to be concentrated in GJCs on the nuclear end of the myelin (abaxonal), whereas Cx29 form HCs was found to be concentrated closer to the paranodal loop and axon (abaxonal). This suggests that they could both contribute to reflexive junctions across the myelin sheath, speeding up communication through the myelin layers that separate the adaxonal and the abaxonal cytoplasm (Figure 1).

## Diseases Associated With Dysfunction of Connexins in Schwann Cells

Charcot-Marie-Tooth disease (CMT) is a large group of disorders caused by different types of mutations in several genes whose protein are expressed in myelin and/or axonal structures within PNS (Lupski, 1999; Kamholz et al., 2000). Usually, the initial symptoms of CMT are distal weakness and muscle atrophy, manifesting with foot drop and pes cavus. Also, sensory symptoms are often present but tend to be less prominent. Later, these patients present foot deformities, such as hammertoes, together with hand weakness and atrophy (Saporta, 2014).

The categories of CMT are CMT types 1 through 7, as well as an X-linked category, CMTX. Then, there are X-linked dominant and recessive forms of CMT involving different loci. Together, the X-linked forms account for 10% to 15% of all CMT cases (Ouvrier et al., 2007). CMTX1, the X-linked dominant form of CMT, is the second most common form of CMT and the first X-linked form of CMT, with 7% to 12%, and 50% of all CMT cases and X-linked cases, respectively (Boerkoel et al., 2002; Huttner et al., 2006; Ouvrier et al., 2007; Pareyson and Marchesi, 2009; Fridman et al., 2015).

CMTX1 symptoms are more prominent in boys, who present gait problems in infancy or later in childhood (e.g., toe walking, flat-footed walking, falls, difficulty running; Yiu et al., 2011). Less common features include tremors, hand weakness, and sensorineural deafness. Reflexes are lost at the ankles in all cases, whereas patellar reflexes are retained in half of the cases in girls. The neuropathy may be asymmetric and therefore mimic an acquired immune-mediated neuropathy (Saporta, 2014). Typically, the pathophysiology of CMTX1 includes features of both demyelination and axon loss, and this is reflected in neurophysiologic studies. Nerve conduction velocities are moderately slowed (Nicholson and Nash, 1993). Demyelination and axonal loss are observed histologically, but onion bulb formation is minimal.

CMTX1 is caused by mutations in the *GJB1* gene, i.e., Cx32 gene, on chromosome Xq13.1 (Hahn et al., 1990; Bergoffen et al., 1993a,b; Keller and Chance, 1999). Most *GJB1* mutations cause disability through the loss of function of Cx32 (Bruzzone et al., 1994; Kleopa et al., 2012). In particular, Cx32 mutations related to CMTX1 could be organized into five classes: (1) Cx32 protein is not synthesized; (2) mutant Cx32 protein has a reduced expression (with a normal transcription), (3) mutant Cx32 protein has a normal expression but is not transported to the plasma membrane; (4) mutant Cx32 protein forms HCs but not functional GJCs; and (5) mutant Cx32 protein forms GJCs and HCs with altered electrical, gating or permeability properties (Bortolozzi, 2018).

It is worth mentioning that some mutants that appear as “functional,” with respect to the WT model, cause severe phenotypes of CMTX1. This issue may be explained by the fact that in most of the studies only the activity of GJCs was evaluated, without considering the gating/permeability dysfunction of mutant Cx32 HCs. Recently, an investigation uncovered that the loss of the C-terminus in Cx32 (R220X mutation), inhibits Cx32 HCs gating. However, it does not significantly alter the intercellular diffusion mediated by GJCs: the unitary permeabilities to ions, signaling molecules (cAMP) or larger solutes (Lucifer yellow) concerning the wild-type (Carrer et al., 2018). These findings support the hypothesis that paracrine signaling alteration, due to Cx32 HCs dysfunction, underlies CMTX1 pathogenesis.

Although it is described that two linked connexons, i.e., a GJCs, may contain the same connexin proteins, or different ones, an interesting case has been described, in myelinated axons, where a Cx29-based connexon is linked to a K^+^ channel (Rash et al., 2016; Traub et al., 2018). This xenotypic channel could be helpful to understand why Cx29 KO mice do not show important neuropathic symptoms, such as Cx32 KO. First, the formation of axolemmal rosettes is an inherent property of K_V_1 channels, does not depend on a linkage with Cx29 (Rash et al., 2016), so it is possible to suggest that in the absence of Cx29 it is still present in “half” of the activity of this xenotypic channel. Second, the absence of the Cx32 protein necessarily involves a total loss of Cx32-based channel activity in myelinated cells, with no compensation from other Cx proteins (Sargiannidou et al., 2009), so the absence of Cx32 would lead to worse results than the absence of Cx29. It was also recently proposed that Cx32 HCs could underlie CMTX1 by altering the purinergic signaling controlling SCs myelination (Nualart-Marti et al., 2013; Carrer et al., 2018). Additionally, if we consider that a heteromeric mixing of Cx29 and Cx32 (i.e., Cx32-Cx29/Cx32-Cx32) produced channels with properties different from those of Cx32 alone (Altevogt et al., 2002), in cases of Cx32 mutations, an unexplored (and speculative) possibility is that these mutations can change the affinity of Cx32 for Cx29 and these “new” connexons could be involved in different failures of Cx32 GJCs activity. The Cx32 protein then becomes an essential protein in the physiology of PNS (Bortolozzi, 2018).

Finally, considering that the current “treatments” include only palliative care, it is necessary to advance new therapies. A recent study obtained a cell-specific expression of Cx32 in up to 50% of SCs, in multiple lumbar spinal roots and peripheral nerves, after a single intrathecal gene delivery into adult *GJB1*-KO mice. Thus, treated mice showed a reduced amount of demyelinated fibers and inflammatory cells with motor performance, quadriceps muscle contractility, and sciatic nerve conduction velocities improved (Kagiava et al., 2016). The same gene therapy was performed in CMT1X mice expressing different Cx32 mutants, which showed different results, as CMT1X mutants may interfere with gene addition therapy (Kagiava et al., 2018). A better approach may be one that does not incorporate the genome but leads to permanent modification of the targeted genetic defect. In this regard, recent reports of treating mouse models of Duchenne muscular dystrophy, with adeno-associated viral vectors, which are not integrated into the genome, is promising. This method used the clustered regularly interspaced short palindromic repeats system (CRISPR)-Cas9 to target and eliminate mutant exons of dystrophin (Nelson et al., 2016; Tabebordbar et al., 2016). In summary, the actual data suggest that gene therapy might become a realistic possibility for patients with these disorders.

## Conclusions

Myelinating SCs and peripheral axons present an interesting example of two specialized cells, where each is indispensable for the functioning of the other. In this sense, the communication through GJCs is a significant evolutionary advance which can solve the issue of the reflexive GJs in the myelinating SCs. This cellular specialization has also led to the differential expression and specific roles of Cx29 and Cx32. In particular, CMTX1 is a clear example of diseases that are caused by mutations in only one GJ gene (Cx32), and despite this broad expression pattern, peripheral neuropathy is the main clinical manifestation. Since several mutations of the Cx32 gene in SCs lead to lasting and severe motor and neurological problems, the search for curative treatment points towards gene therapies in an attempt to replace the defective gene. These approaches however, have their limitations because of toxicity from random integrations in the genome.

## Author Contributions

CP and BC conceived, researched and wrote this review article. PA assisted in writing and editing.

## Conflict of Interest Statement

The authors declare that the research was conducted in the absence of any commercial or financial relationships that could be construed as a potential conflict of interest.
